# A moderated chain mediation model examining the relation between smartphone addiction and intolerance of uncertainty among master's and PhD students

**DOI:** 10.1016/j.heliyon.2024.e30994

**Published:** 2024-05-10

**Authors:** Huake Qiu, Hongliang Lu, Xianyang Wang, Zhihua Guo, Chen Xing, Yan Zhang

**Affiliations:** Department of Military Medical Psychology, Air Force Medical University, No. 169 West Changle Road, Xi'an, Shaanxi Province, 710032, China

**Keywords:** Intolerance of uncertainty, Anxiety, Positive coping style, Perceived social support, Smartphone addiction

## Abstract

The theories of relational regulation and compensatory Internet use suggest that intolerance of uncertainty influences smartphone addiction (SPA), which in turn is influenced by other aspects. This study used previous results to examine how intolerance of uncertainty affects SPA in PhD and master's degree programs. A convenience sample comprising 1727 master's and PhD students (99.9 %; 50.7 % female; *M*_*age*_ = 27.71; *SD* = 3.80; range = 21–43) was recruited. Using established questionnaires, we measured SPA, anxiety, positive coping style, perceived social support, and intolerance of uncertainty. The results demonstrated that intolerance of uncertainty positively affects SPA in terms of predictive power. Furthermore, anxiety and positive coping style mediate that link in a cascade fashion. A greater SPA indicates that the individual is less likely to use a positive coping style, has a higher anxiety level, and has a lower tolerance for uncertainty. While thinking about how intolerance of uncertainty affects anxiety and positive coping style, perceived social support mediates the relationship. Intolerance of uncertainty has less impact on anxiety and positive coping style when perceived social support is high. These results indicate the possibility of examining SPA prevention and intervention from several angles. Therefore, emotional regulation, which modifies anxiety and the tendency to use a positive coping style, may reduce the impact of intolerance of uncertainty on SPA. Another successful strategy for reducing smartphone addiction is to provide social support from loved ones and the community at large.

## 1 Introduction

Due to the progress of science and technology, the smartphone has evolved from a basic means of communication into an essential component of everyday existence. In addition to relieving some of the negative impacts of stress, several studies have shown that people form strong emotional bonds with smartphones. Consequently, individuals will experience feelings of distress when they are briefly unable to use their smartphones [[1], [2], [3], [4]]. The phenomenon of experiencing anxiety due to the lack of access to a smartphone is examined within the framework of smartphone separation anxiety [2,5].

The smartphone is a good way for people to use the Internet; thus, it has a positive impact to some degree. Smartphone use to support communication helps to strengthen the connection between individuals and society [6] and has a positive effect on mental health, which helps to alleviate the influence of stressful life events [7,8]. Nevertheless, smartphone addiction (SPA), which develops from heavy smartphone use, has emerged as a complicated and growing public health concern, particularly among youth [9,10]. Addiction to smartphones is considered a behavioral addiction, implying that despite the considerable adverse effects of smartphone use, individuals continue to engage in excessive and uncontrolled smartphone usage [[11], [12], [13]]. SPA has been associated with psychological problems [14]. Furthermore, a comprehensive analysis has revealed that increased smartphone use is related to poor academic performance [15]. This is due to the correlation between SPA and students' sleep quality, which subsequently impacts their academic achievement [16]. Master's and PhD students have higher levels of research and life stress than undergraduates and may also exhibit a greater reliance on smartphones. Consequently, this study chose master's and PhD students as participants to investigate the factors that influence SPA.

### 1.1 Intolerance of uncertainty and SPA

A cognitive bias known as intolerance of uncertainty influences how people interpret, process, and react to uncertain circumstances [[17], [18], [19]]. It causes people to view ambiguous situations in a negative light, which in turn causes them to react negatively, for example, by avoiding them. The idea of compensatory Internet use states that when people experience bad things in life, they are driven to respond by using their smartphones to alleviate their suffering [20,21]. When people react negatively to situations or occurrences that are unknown, it's called intolerance of uncertainty [17]. The anxiety experienced in ambiguous circumstances can be alleviated by browsing the Internet or utilizing smartphones. Smartphone usage is said to function as an "adult pacifier," offering psychological solace and alleviating the adverse emotions associated with regular stress [22]. According to a meta-analysis [23], smartphone usage is crucial in Internet addiction and future expectations [24]. Researchers found that a person's intolerance of uncertainty mediated the relationship between their capacity to control their emotions and their fear of being unable to use their smartphone [25]. People who have a strong aversion to uncertainty and engage in excessive usage of smartphones may develop SPA, especially if they also have cyberchondria [26].

The effects of uncertainty intolerance on SPA and anxiety are substantial in this study. However, few research examined how social anxiety and intolerance of uncertainty are related in PhD and master's degree candidates. Hence, hypothesis 1 is proposed..

H1 Both master's and doctoral students are at increased risk for SPA if they are intolerant of uncertainty. There is a correlation between high SPA and an elevated intolerance of uncertainty.

### 1.2 Anxiety and positive coping style

Anxiety is a prevalent psychological disorder [27]. Concerns about what lies ahead can result in distress and unease, and a lack of tolerance for unpredictability contributes to anxiety disorders [28]. Intolerance of uncertainty is a key cognitive component that predisposes individuals to generalized anxiety disorder (GAD) and anxiety, and it is one of several critical cognitive aspects that promote the development of various anxiety disorders [[29], [30], [31], [32]]. Most prior research on SPA and psychopathology has relied on bivariate and regression analysis [[33], [34], [35], [36], [37]]. Previous research on anxiety has shown a strong positive correlation between anxiety and SPA [[38], [39], [40], [41]]. Coping style refers to individuals' customary tactics when confronted with various events and situations [42]. Researchers examined the link between coping style and intolerance of uncertainty and found a significant correlation between them [43,44]. According to Yao et al., intolerance of uncertainty is associated with adopting negative coping strategies [44]. Furthermore, positive coping style influences the connection between intolerance of uncertainty and SPA [45]. The relationship between various coping strategies and SPA is considerable. Specifically, problem-centered coping has been identified as a crucial component contributing to SPA in children and adolescents [46]. Thus, both anxiety and a positive coping style have a strong influence on SPA, and they act as a mediator between intolerance of uncertainty and SPA.

Anxiety is strongly correlated with various coping strategies [47,48]. The presence of a negative coping style exacerbates the persistence of worries and subsequently contributes to anxiety and depression [44,49]. As a protective factor, a positive coping style reduces the effects of anxiety and shows a negative correlation with anxiety [[50], [51], [52]]. These studies demonstrate a strong correlation between anxiety and positive coping style. Furthermore, it is worth noting that individuals may employ various coping strategies, particularly in cases of depression and anxiety [53]. Consequently, the varying levels of despair or anxiety experienced by individuals may be attributed to their utilization of distinct coping strategies. The relationship between SPA and intolerance of uncertainty is moderated sequentially by anxiety and positive coping style. Therefore, this study puts forward hypothesis 2.

H2 Interactions between intolerance of uncertainty and SPA in PhD and master's students are mediated by anxiety and positive coping style. The development of SPA is facilitated by an increased intolerance of uncertainty, which in turn increases anxiety levels and decreases the use of positive coping style. Worrying too much also makes it difficult to use positive coping style, which makes SPA worse.

### 1.3 Perceived social support

Intolerance of uncertainty causes individuals to lose their sense of control in uncertain situations, which can result in maladaptive psychological responses like anxiety. Furthermore, during the recent pandemic, anxiety levels were found to be significantly influenced by how much social support individuals felt. Specifically, higher levels of social support correlate with decreased anxiety [[54], [55], [56]]. A robust protective factor against anxiety and depression was determined by Roohafza et al. to be the perception of social support, particularly from family members [52]. Perceived social support is inversely related to anxiety. More social support is required to reduce anxiety [57]. Perceived social support also effectively reduces the effect of several situations on anxiety in college students [58,59]. Therefore, social support is key to protecting people's mental health [[60], [61], [62]].

Perceived social support is a valuable asset for dealing with difficult situations, as it assists in altering perceptions of uncertain occurrences and enhancing coping skills [63,64]. Chinese immigrants who firmly adhere to Chinese values experience more perceived social support, which increases their likelihood of using a positive coping method [65]. Furthermore, we may anticipate occupational burnout among Chinese firefighters by analyzing their coping mechanisms based on their perceptions of social support [66]. Researchers showed that [67,68], a good coping style influences the link between perceived social support and post-traumatic growth. Good coping strategies are also predicted by how much social support one feels they possess.

Lakey and Orehek (2011) proposed relational regulation theory (RRT) as a theoretical framework. As per the Regulation of Emotion, Thought, and Behavior (RRT) theory, individuals manage their emotions, thoughts, and behaviors by engaging in social interactions. The manifestation of this conversation in social interactions is primarily observed in everyday situations rather than in high-pressure circumstances [69,70]. Hence, the perception of social support can safeguard against intolerance of uncertainty, alleviate anxiety, and promote the adoption of a positive coping style. We thus proposed the following hypothesis 3.

H3 Perceived social support regulates the mediating mechanism among master's and PhD students. Thus, SPA is influenced by regulates perceived social support, which in turn impacts the relationship between anxiety, intolerance of uncertainty, and positive coping style. When individuals experience strong social support, their anxiety and positive coping style are less affected by intolerance of uncertainty, resulting in lower levels of SPA.

**Fig. 1:**
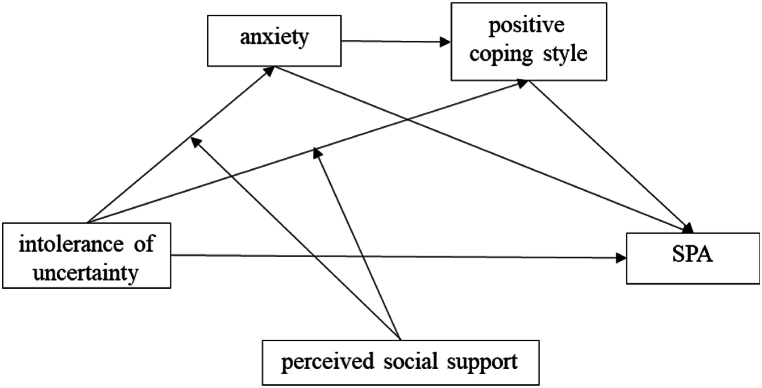
Hypothetical model.

No research has been conducted on the correlation between intolerance of uncertainty and SPA among master's and PhD students. Furthermore, anxiety and coping style have reciprocal effects on each other. Existing studies mostly emphasize the significant impact of negative coping style but have not conducted much study on positive coping style, which are as important. Positive psychological features of individuals have been found to have a crucial function in safeguarding mental health, according to positive psychology [71]. Therefore, this study aims to recruit master's and PhD students to investigate the relationship between SPA and intolerance of uncertainty, focusing on the mediating roles played by anxiety and positive coping strategies. In addition to being an important factor in deciding how to cope, the feeling of social support is a major factor in protecting against anxiety. Thus, this study chooses perceived social support to examine its role in moderating the relationship. Fig. 1 presents the proposed hypothetical model.

## 2 Method

### 2.1 Participants and ethics

Students pursuing master's and doctoral degrees in northwest China were surveyed using a questionnaire. The survey was carried out over approximately six months using convenience sampling. The experimenters were tasked with delivering the surveys using computer terminals, and the participants were arranged in the computer room to complete the questionnaires. A total of 1728 surveys were distributed, with one questionnaire being excluded due to missing data. The data used in this analysis came from 1727 people, with 99.9 % male and 49.3 % female. The participants' ages varied from 21 to 43 years, averaging 27.71 ± 3.80 years. Table 1 depicts the comprehensive demographic data. The Ethics Committee of Xijing Hospital has examined and approved this study (Ethics Committee approval number: KY20202063–F-2). All participants gave their "informed consent" to participate by signing the consent forms.

**Table 1:** Demographic data of the participants.

Variables		n (%) or *M* ± *SD*
Gender	Male	851 (49.3 %)
	Female	876 (50.7 %)
Education	Master's	1352 (78.3 %)
	Doctorate	375 (21.7 %)
Major	Medicine	1127 (65.3 %)
	Psychology	123 (7.1 %)
	Pharmacy	79 (4.6 %)
	Biology	94 (5.4 %)
	Pedagogy	304 (17.6 %)
Age (years)		27.71 ± 3.80
Enrollment time (years)		2.18 ± 1.71

### 2.2 Measures

#### 2.2.1 Intolerance of uncertainty scale

The IUS was created to quantify the level of intolerance of uncertainty with high internal consistency reliability and structural validity levels [72]. The scale consists of twelve components assessed on a five-point Likert scale, where 1 indicates never and 5 indicates extremely. The level of intolerance for uncertainty is directly proportional to the score. The IUS was tested for validity and reliability after being adjusted [73] to fit China's unique circumstances [47,74]. Cronbach's *α* coefficient was found to be 0.88.

#### 2.2.2 Smartphone addiction scale -short version

The SAS-SV showed excellent concurrent validity and internal consistency reliability. It was created to assess SPA [75,76]. Each item has a six-point rated scale, where 1 indicates strong agreement, and 6 indicates strong disagreement; participants assess their level of consent according to each item. The likelihood of SPA increases as the score rises. In this case, Cronbach's *α* was 0.90.

#### 2.2.3 Generalized anxiety disorder

Spitzer et al. [77] developed the GAD-7, which has extensively evaluated people's mental health [78]. It comprises 7 seven items. A four-point rated scale, from 0 *(never)* to 3*(nearly every day)*, is used to evaluate each item. The more anxious you are, the higher the score. The possible sums are between zero and twenty-one. A GAD score of 10 is considered significant. Using this survey with a wide range of demographics has shown its reliability and validity to be very high [77,79]. Cronbach's *α* for this scale was 0.90.

#### 2.2.4 Simplified coping style questionnaire

Based on ways of coping questionnaire, the SCSQ [80] was used to evaluate positive coping style [81]. It comprises 20 items that are four-point rated from 0 *(never)* to 3 *(very often)* and is composed of two sub-questionnaires. The positive coping style sub-questionnaire comprises 12 items, like “Release through work, study or some other activity.” The negative coping style sub-questionnaire comprises eight items, such as “smoke, drink, take medication, and eat to relieve the worry.” The reliability and validity of the questionnaire used in the initial investigation [80] were very high. It has been regularly validated and relied upon by the general Chinese, and its use has been widespread [82]. The positive coping style was the sole focus of this investigation. In this case, Cronbach's *α* was 0.94.

#### 2.2.5 Perceived social support scale

Overall, perceived social support was the goal of developing the PSSS [83,84]. According to research by Zimet et al. (1988), the scale had high levels of structural validity and internal consistency. The 12-item measuring scale includes statements like, "I can talk to my friends about my issues." Each item has a 7-point scale, with 1 being very disagree and 7 representing very agree. Further, a higher score indicates that people feel they have more social support. This scale had a Cronbach's *α* of 0.96.

### 2.3 Procedure

A specific online link was distributed to participants simultaneously. Once the link was clicked, the informed consent page appeared. The page explained participants' anonymity and confidentiality of the results while explaining the study's objective. Participants initially provided their demographic information during the questionnaires. They completed the IUS, GAD-7, SCSQ, and SAS-SV. Finally, the participants filled out the PSSS.

### 2.4 Statistical analyses

We used SPSS 28.0 to analyze all of the data. Descriptive statistics and correlation analysis were used to determine the relationships between the variables. Afterward, the PROCESS macro (model 6) was used to evaluate the chain mediation model [85], which aimed to determine if anxiety and positive coping style mediated the association between intolerance of uncertainty and SPA. When the time came to study how perceived social support moderated the chain mediation model, the PROCESS macro (model 84) was employed [85].

## 3 Results

### 3.1 Common method bias test

We used Harman's single-factor test to examine the potential common method bias using self-reported questionnaires. Harman's unrotated revealed that ten components were considered, with an eigenvalue greater than 1. The first factor considers less than 40 % of the total variance, which is 26.04 %. Hence, common method bias did not significantly impact the outcomes.

### 3.2 Descriptive statistics and correlation analysis

Table 2 depicts the results of the analysis performed on the study variables. Statistical analysis revealed a positive association between SPA and intolerance of uncertainty. Anxiety, SPA, and intolerance of uncertainty were all negatively correlated with positive coping style and perceived social support.

**Table 2:** Descriptive statistics and correlations for all variables.

Variable (Score Range)	*M*	*SD*	1	2	3	4	5
intolerance of uncertainty(12–60)	32.95	8.50	1				
SPA(10–60)	24.44	10.00	0.44^a^	1			
anxiety(0–21)	2.38	3.16	0.43^a^	0.40^a^	1		
positive coping style(0–36)	26.48	7.69	−0.26^a^	−0.26^a^	−0.35^a^	1	
perceived social support(12–84)	65.20	11.88	−0.25^a^	−0.23^a^	−0.36^a^	0.60^a^	1

Note: *M* = mean. *SD* = standard deviations.

a *p* < 0.001.

### 3.3 Mediating effect test

We examined how anxiety and positive coping style affect the link between intolerance of uncertainty and SPA using the PROCESS macro (Model 6) to figure out how this trait affects SPA. Table 3 depicts the details of the data used in the analysis. The data demonstrated a significant impact of intolerance of uncertainty on SPA (*β* = 0.44, *t* = 20.42, *p* < 0.001). Considering the mediating variables, it was found that intolerance of uncertainty had a negative effect on positive coping style (*β* = −0.13, *t* = −5.13, *p* < 0.001), a positive effect on anxiety (*β* = 0.43, *t* = 19.72, *p* < 0.001), and a positive effect on SPA (*β* = 0.32, *t* = 13.82, *p* < 0.001). Anxiety negatively predicted positive coping style (*β* = −0.30, *t* = −12.00, *p* < 0.001) and positively predicted SPA (*β* = 0.22, *t* = 9.31, *p* < 0.001). Positive coping style negatively predicted SPA (*β* = −0.10, *t* = −4.37, *p* < 0.001).

**Table 3:** Mediation analysis.

Regression equation		Overall fitting index			Regression coefficient	
Outcome variable	Predictive variable	*R*	*R*^*2*^	*F(df)*	*β*	*t*
anxiety		0.43	0.18	388.98_(1)_^a^		
	intolerance of uncertainty				0.43	19.72^a^
positive coping style		0.37	0.14	136.72_(2)_^a^		
	intolerance of uncertainty				−0.13	−5.13^a^
	anxiety				−0.30	−12.00^a^
SPA		0.44	0.19	416.88_(1)_^a^		
	intolerance of uncertainty				0.44	20.42^a^
SPA		0.50	0.25	196.35_(3)_^a^		
	intolerance of uncertainty				0.32	13.82^a^
	anxiety				0.22	9.31^a^
	positive coping style				−0.10	−4.37^a^

Note: All variables in the model were entered into the regression equation after standardization.

a *p* < 0.001.

We used a bias-corrected bootstrap test with 5000 samples and a 95 % confidence interval to check whether the mediating effects were statistically significant. Table 4 depicts all the data of the analysis. A significant total effect was found linking intolerance of uncertainty to SPA (*β* = 0.441, *SE* = 0.022, *95 % CI* = 0.399–0.484). Both anxiety (*β* = 0.096, *SE* = 0.013, *95 % CI* = 0.072–0.122) and positive coping style (*β* = 0.012, *SE* = 0.003, *95 % CI* = 0.005–0.022) had a significant indirect effect. The results also show a significant chain mediating effect of anxiety and positive coping style (*β* = 0.013, *SE* = 0.003, *95 % CI* = 0.006–0.019).

**Table 4:** Examining the paths of the mediation model.

		β	SE	*95 % confidence interval*	
				Lower	Upper
total effect		0.441	0.022	0.399	0.484
direct effect		0.321	0.023	0.275	0.366
indirect effect		0.121	0.013	0.095	0.148
	Indirect 1	0.096	0.013	0.072	0.122
	Indirect 2	0.012	0.004	0.005	0.022
	Indirect 3	0.013	0.003	0.006	0.019

Note: Indirect 1: intolerance of uncertainty - > anxiety - > SPA; Indirect 2: intolerance of uncertainty - > positive coping style - > SPA; Indirect 3: intolerance of uncertainty - > anxiety - > positive coping style - > SPA.

### 3.4 Test of moderated chain mediating effect

We used the PROCESS macro (Model 84) to create a moderated chain mediation model to examine the role of perceived social support. Table 5 presents all the data of the analysis. Intolerance of uncertainty and perceived social support was a significantly negative predictor of anxiety (*β* = −0.06, *SE* = 0.02, *t* = −3.33, *p* < 0.001, *95 % CI* = −0.09 to −0.02) and positive coping style (*β* = −0.04, *SE* = 0.02, *t* = −2.50, *p* < 0.05, *95 % CI* = −0.07 to −0.01).

**Table 5:** Analysis of moderated mediation.

Regression equation		Overall fitting index			Regression coefficient	
Outcome variable	Predictive variable	*R*	*R*^*2*^	*F(df)*	*β*	*t*
anxiety		0.51	0.26	198.78_(3)_^c^		
	intolerance of uncertainty				0.35	16.45^c^
	perceived social support				−0.28	−12.83^c^
	perceived social support^a^ intolerance of uncertainty				−0.06	−3.33^c^
positive coping style		0.62	0.39	275.75_(4)_^c^		
	intolerance of uncertainty				−0.06	−3.06^b^
	anxiety				−0.13	−6.13^c^
	perceived social support				0.54	26.24^c^
	perceived social support^a^ intolerance of uncertainty				−0.04	−2.50^a^
SPA		0.50	0.25	196.35_(3)_^c^		
	intolerance of uncertainty				0.32	13.82^c^
	anxiety				0.22	9.31^c^
	positive coping style				−0.10	−4.37^c^

Note: All variables in the model were entered into the regression equation after standardization.

a *p* < 0.05.

b *p* < 0.01.

c *p* < 0.001.

**Fig. 2:**
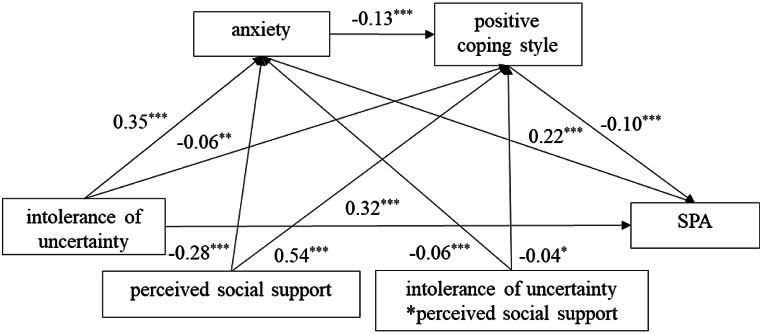
Statistical model.

According to the results shown in Table 6, perceived social support played a moderating role between intolerance of uncertainty and SPA. Low perceived social support (Indirect 1: *β* = 0.092, *SE* = 0.014, *95 % CI* = 0.065–0.121; Indirect 3: *β* = 0.005, *SE* = 0.002, *95 % CI* = 0.002–0.009) was more predictive of SPA than high perceived social support (Indirect 1: *β* = 0.066, *SE* = 0.010, *95 % CI* = 0.047–0.087; Indirect 3: *β* = 0.004, *SE* = 0.001, *95 % CI* = 0.002–0.007). Positive coping style (Indirect 2) had a significant mediating effect under high perceived social support (*β* = 0.010, *SE* = 0.004, *95 % CI* = 0.003–0.018), but not under low perceived social support (*β* = 0.003, *SE* = 0.003, *95 % CI* = 0.002–0.009). Fig. 2 depicts the statistical model of this study.

**Table 6:** The impact of different levels of perceived social support on moderation.

indirect effect		*β*	*SE*	*95 % confidence interval*	
				Lower	Upper
Indirect 1	high perceived social support	0.066	0.010	0.047	0.087
	low perceived social support	0.092	0.014	0.065	0.121
Indirect 2	high perceived social support	0.010	0.004	0.003	0.018
	low perceived social support	0.003	0.003	−0.003	0.009
Indirect 3	high perceived social support	0.004	0.001	0.002	0.007
	low perceived social support	0.005	0.002	0.002	0.009

Note: Indirect 1: intolerance of uncertainty - > anxiety - > SPA; Indirect 2: intolerance of uncertainty - > positive coping style - > SPA; Indirect 3: intolerance of uncertainty - > anxiety - > positive coping style - > SPA.

## 4 Discussion

In contemporary society, the widespread popularity of smartphones has made people rely more on their smartphones when uncertain situations emerge, which can lead to SPA. The present investigation focused on intolerance of uncertainty, SPA, and psychological mechanisms. According to the hypothesis, anxiety and positive coping style are thought to mediate the link between intolerance of uncertainty and SPA. Perceived social support moderates the impact of intolerance of uncertainty on anxiety and positive coping style.

### 4.1 Intolerance of uncertainty and SPA

The descriptive statistical data indicate that the average score of SPA falls below the critical value for SPA (Male: 31; Female: 33). This could be attributed to the specific demographic of participants chosen for this study. This study specifically recruited master's and PhD students who did not have SPA, resulting in a participant pool consisting mostly of individuals with good physical and mental health. The scale scores alone indicate the extent of SPA among different participants, making it challenging for the average scale score to meet the necessary threshold for SPA.

According to our findings, high intolerance of uncertainty leads to high SPA, which is consistent with current studies. This result confirms hypothesis 1. A recent study found that an intolerance of uncertainty mediates the link between chronic stress and SPA [13]. Another study [86] found that intolerance of uncertainty strongly predicts problematic smartphone use a month later. Both high intolerance of uncertainty and cyberchondria have been found to predict SPA [26]. Moreover, the emotional response to uncertainty is the key reason SPA results from intolerance of uncertainty [87]. One possible explanation for people's negative emotional reactions to adverse occurrences is that they utilize the Internet or smartphones to compensate [21]. This finding further confirms the compensatory Internet use theory.

### 4.2 Anxiety and positive coping style

Anxiety, SPA, and intolerance of uncertainty were found to be positively related in this study using correlation analysis. These characteristics were found to have a negative correlation with positive coping style. Thus, the present study intended to construct a mediation model to examine the mediating roles of anxiety and positive coping style. Therefore, the present study built a model to investigate mediating roles of anxiety and positive coping style. Consistent with earlier studies, our findings showed that intolerance of uncertainty affects SPA through the anxiety above and positive coping style. The effects of intolerance of uncertainty on anxiety [28,[88], [89], [90]] and positive coping style [49,89,91] have been extensively documented. It is also believed that intolerance of uncertainty is a transdiagnostic sustaining factor across depressive and anxiety disorders [88,92]. Furthermore, researchers showed that varying degrees of intolerance of uncertainty have distinct coping mechanisms, consistent with our study's findings [93,94].

Anxiety and positive coping style mediate the relationship between the two, according to our results. A person's SPA may alter as a result of their intolerance of uncertainty, affecting their anxiety and positive coping style. However, previous research on the correlation between SPA and intolerance of uncertainty has not specifically examined the role of anxiety and positive coping style as mediators. Nonetheless, other studies have found evidence that lends indirect support to the findings of this one. Prior research has indicated that various coping mechanisms are associated with anxiety to varied degrees [95]. A variety of coping styles are employed by individuals experiencing varying degrees of anxiety and depression [53]. Furthermore, the predictive power of positive coping style for anxiety has been the subject of a few research [42,96]. Nevertheless, the I-PACE model [97] suggests that components including emotion, cognition, and executive function are significant in predicting addictive behavior. Both the individual effects and the synergies between these variables contribute to the development of addiction. Thus, we hypothesized that anxiety levels influence coping style selection; indeed, our findings demonstrate that anxiety levels predict positive coping style selection and that these style mediate the association between intolerance of uncertainty and SPA.

### 4.3 Perceived social support

We found that perceived social support negatively moderates the impact of intolerance of uncertainty on anxiety and positive coping style, consistent with previous research. A significant correlation was obtained between perceived social support, anxiety [98,99], and positive coping style [67]. Besides, perceived social support can predict changes in anxiety [100] and coping style [101]. According to RRT, individuals regulate their thoughts, emotions, and behavior in social communication [70]. Simultaneously, based on the I-PACE model, it is suggested that the interaction between different factors also has an impact on addiction behavior [97]. Under high perceived social support, intolerance of uncertainty leads to less anxiety and more use of positive coping style. This may be because higher perceived social support makes individuals calmer and more tolerant in the face of uncertainty, thus reducing anxiety and causing them to adopt a more positive coping style. Therefore, this study extends prior research on SPA and finds a protective role for perceived social support in the mediating relationship.

### 4.4 Limitations and future directions

This study is primarily based on research type and data collection method. First, a cross-sectional study was conducted to investigate the impact of intolerance of uncertainty on SPA and its psychological process. Researchers examined multiple factors simultaneously in a cross-sectional study and found their relationship [102]. However, it is difficult to assess how factors relate to one another over time and to establish whether intolerance of uncertainty long-term impacts SPA. Hence, future studies must assess the influence on the temporal dimension thoroughly. To mitigate the influence of certain periods on the data, it is recommended to gather study data repeatedly at various intervals.

Second, considering the difficulty of offline data collection with master's and PhD students, this study adopted online data collection instead of the traditional face-to-face collection. Although online data acquisition is convenient, it cannot scrutinize participants' responses (for example, it cannot determine whether answers are intended seriously), which may affect the data's validity. To maintain the reliability of the data, future studies should implement measures to regulate the total number of items collected in online data collection. Additionally, it is advisable to employ appropriate statistical methods for data analysis.

### 4.5 Implications

This study has certain crucial implications. Focusing on the theoretical implications, this is the first study to assess the impact of intolerance of uncertainty on SPA utilizing a sample of master's and PhD students. Master's and PhD students have higher academic pressure than college students and may also encounter additional personal pressures like beginning a family. Consequently, they are expected to rely more heavily on cell phones. However, studies investigating the factors impacting SPA among PhD and master's degree candidates are still in the early stages. This study establishes a foundation for future investigations on the self-perceived academic performance of master's and PhD candidates.

Perceived social support, anxiety, and positive coping style affect the relationship between intolerance of uncertainty and SPA. It suggests various methods to decrease the SPA of master's and PhD students. Emotional control and other techniques effectively decrease anxiety, encouraging pupils to adopt a positive coping style and further suppress SPA. Conversely, perceived social support safeguards master's and PhD students. This implies that individuals can decrease their smartphone usage by enhancing their perception of social support, which holds practical importance.

## 5 Conclusion

In summary, recent studies found that intolerance of uncertainty affected SPA and built a model of that relationship based on moderated chain mediation. According to our study, intolerance of uncertainty strongly correlates with SPA. The degree of anxiety and the presence or absence of positive coping style impact this relationship. In addition, our results show that the impact of the relationship was governed by how people perceived their social support system. Anxiety and positive coping style mediated the relationship between intolerance of uncertainty and SPA, according to our results. According to our findings, perceived social support moderated the relationship's mediation. A decreased impact of intolerance of uncertainty on anxiety and positive coping style, leading to a reduction in SPA, was observed in conditions of high perceived social support. Research and clinical practice can benefit from this study's findings by delving further into the potential therapeutic or preventative effects of increasing perceived social support on decreasing SPA among PhD and master's degree students.

## Data availability statement

The data that has been used is confidential, so supporting data is not available.

## Ethics statement

This study was reviewed and approved by Xijing Hospital, with the approval number: KY20202063–F-2.

## Project funding

"Quick Response" Research Project of AFMU (2022KXKT014)

## CRediT authorship contribution statement

**Huake Qiu:** Writing – review & editing, Writing – original draft, Software, Investigation, Conceptualization. **Hongliang Lu:** Writing – review & editing, Supervision, Investigation, Conceptualization. **Xianyang Wang:** Supervision, Investigation. **Zhihua Guo:** Software, Investigation. **Chen Xing:** Writing – review & editing, Supervision, Software. **Yan Zhang:** Writing – review & editing, Supervision.

## Declaration of competing interest

The authors declare that they have no known competing financial interests or personal relationships that could have appeared to influence the work reported in this paper.
